# Information Discovery on Electronic Health Records Using Authority Flow Techniques

**DOI:** 10.1186/1472-6947-10-64

**Published:** 2010-10-22

**Authors:** Vagelis Hristidis, Ramakrishna R Varadarajan, Paul Biondich, Michael Weiner

**Affiliations:** 1 School of Computing and Information Sciences, Florida International University, Miami, Florida, USA; 2 Department of Computer Sciences, University of Wisconsin-Madison, Wisconsin, USA; 3 Regenstrief Institute, Inc., Indianapolis, Indiana, USA; 4 Indiana University School of Medicine, Indianapolis, Indiana, USA; 5 Indiana University Center for Health Services and Outcomes Research, Indianapolis, Indiana, USA; 6 VA HSR&D Center of Excellence on Implementing Evidence-Based Practice, Richard L. Roudebush VA Medical Center, Indianapolis, Indiana, USA

## Abstract

**Background:**

As the use of electronic health records (EHRs) becomes more widespread, so does the need to search and provide effective information discovery within them. Querying by keyword has emerged as one of the most effective paradigms for searching. Most work in this area is based on traditional Information Retrieval (IR) techniques, where each document is compared individually against the query. We compare the effectiveness of two fundamentally different techniques for keyword search of EHRs.

**Methods:**

We built two ranking systems. The traditional BM25 system exploits the EHRs' content without regard to association among entities within. The Clinical ObjectRank (CO) system exploits the entities' associations in EHRs using an authority-flow algorithm to discover the most relevant entities. BM25 and CO were deployed on an EHR dataset of the cardiovascular division of Miami Children's Hospital. Using sequences of keywords as queries, sensitivity and specificity were measured by two physicians for a set of 11 queries related to congenital cardiac disease.

**Results:**

Our pilot evaluation showed that CO outperforms BM25 in terms of sensitivity (65% vs. 38%) by 71% on average, while maintaining the specificity (64% vs. 61%). The evaluation was done by two physicians.

**Conclusions:**

Authority-flow techniques can greatly improve the detection of relevant information in EHRs and hence deserve further study.

## Background

Improving the quality and safety of care often requires identification of medical records that meet specified criteria. Although the availability of electronic administrative and clinical data has facilitated many types of automated searches, the searching often requires technical expertise, slow batch processes, and tolerance for low sensitivity of results. The Nationwide Health Information Network and its data-sharing building blocks, Regional Health Information Organizations in the United States, are encouraging the widespread adoption of electronic health records (EHRs) for all hospitals by 2014. As the use of EHRs becomes more widespread, so does the need to search and provide effective information discovery within them. Querying by keyword has emerged as one of the most effective paradigms for searching. Most work in this area is based on traditional Information Retrieval (IR) techniques, where each document is compared individually against the query. More sensitive, complete, systematic methods to discover information in EHRs may enable practitioners, researchers, safety officers, and other healthcare stakeholders to improve care.

The key focus of information retrieval (IR) is determining how to rank the documents of a collection according to their "goodness" with respect to a query. EHRs are typically complex structured documents containing several associated clinical entities (e.g., physicians, medications, patients, and events). The common query is usually expressed as a list of keywords, similarly to the case of Web search. Other types of queries are possible, but the work described herein relies on queries defined as sequences of keywords. The goodness of a result depends on factors like relevance to the query, specificity, and importance. The relevance is a subjective judgment and may include being about the intended subject, being timely (recent information), being authoritative (from a trusted source) and satisfying the users' goals and their intended uses of the information (information need) [1]. The simplest notion of relevance could be defined as a query string that appears verbatim in a document. In a slightly less strict notion, the words in the query appear frequently in the document, in any order ("bag of words"). The importance of a result is determined by its authoritativeness (should be from a trusted source). The specificity of a result is determined by its relevance (being on the proper subject and satisfying user goals) and conciseness. The ranking factors in IR are generally combined using a ranking function to assign a score to each document. The documents or links to them are then output in decreasing order of their IR score.

Traditional IR ranking methods, like Okapi BM25 [2], ignore the associations among entities and instead view each entity as an individual, disconnected document. To improve the quality of search, techniques recently created for searching the hyperlinked Web exploit associations among entities (e.g., hyperlinks between Web pages). We refer to these techniques as *authority flow ranking *techniques, since the authority (importance) "flows" along associated entities.

We sought to compare the effectiveness of two fundamentally different techniques for keyword search on EHRs: the traditional BM25 technique and a newly proposed authority flow ranking technique, Clinical ObjectRank (CO). CO exploits associations among clinical entities, to compute top-*k *results for keyword queries. Our hypothesis is that the effective use of the associations among clinical entities (patient, hospitalization, clinical test, and so on) in EHRs can improve the quality of search in EHRs. Before describing our methods, we provide additional background details about searching.

## Methods

### Modern Information Retrieval

IR is the science of searching for relevant information in a collection of documents. An IR process begins with a user submitting a query to the IR system, which compares the query to the documents and returns a ranked list of relevant documents. Most common factors used in IR to rank the documents are term frequency (*tf*), inverse document frequency (*idf*), and document length (*dl*) [3]. In *tf*, words that occur multiple times in a document are considered salient. In *idf*, words that appear in many documents are considered "common denominators" and do not especially indicate document content. In *dl*, when collections have documents of varying lengths, longer documents tend to score higher since they contain more words and word repetitions. Normalizing for document lengths in the term weighting method usually compensates this effect.

Most IR formulas combine *tf*, *idf*, and *dl*, to assign a unique score to each document given a query. *Okapi weighting *[4] and *pivoted normalization weighting *[5] are widely used weighting methods. Many IR researchers currently use a variant of these two weightings. Many studies use the phrase *tf-idf weighting *to refer to any term weighting method that uses *tf *and *idf*.

**Figure 1:**
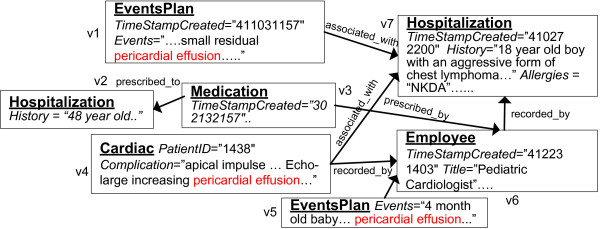
**A subset of Electronic Health Record Dataset**. A subset of the relational-anonymized experimental EHR dataset of the Cardiac Division of Miami Children's Hospital, which contains clinical entities like hospitalization, patient, employee, medication, diagnosis, diagnostics, cardiac, events, and labs.

For example, consider a query "*pericardial effusion*" over the clinical dataset shown in Figure 1. Assume that the ranking function is the product of *tf *and *idf*, i.e., *tf·idf*. In most proposed formulas, the two factors are typically combined by product for better results. By multiplying the two factors, the effect of term frequency is adjusted according to its importance in judging relevance (measured by inverse document frequency (*idf*)). For example, the word "*old*" which appears quite frequently would have a high term frequency but a very low inverse document frequency and hence is not a good term for judging document relevance. Hence it would given a low *tf·idf *score. For example, assuming a collection of 100 medical entities (only a subset of which is shown in the figure) in which the term "*pericardial*" appears in 10 entities, we get *idf*(*pericardial*) = log(100/10) = 1.0. Assuming "*effusion*" appears in 5 documents, we get *idf*(*effusion*) = log(100/5) = 1.30. Since the two terms co-occur, their term frequencies are the same: *tf*(*pericardial*) = *tf*(*effusion*) = 3. This is because *"pericardial effusion" *typically occurs as a phrase. But the term frequency in general is per term frequency, which, in this case, is the same for both terms *"pericardial" *and *"effusion"*. The final *tf·idf *weight of document *v1 *in Figure 1 is then *tf*(*pericardial*)·*idf*(*pericardial*) + *tf*(*effusion*)·*idf*(*effusion*) = (3)·(1.0) + (3)·(1.30) = 6.90.

Okapi BM25 [2] is a state-of-the-art retrieval function used in document retrieval, which is a bag-of-words retrieval function that ranks a set of documents based on the query terms appearing in each document, regardless of the relationship between the query terms within a document (e.g., their relative proximity). BM25 is not a single function but is a family of scoring functions, with various components and parameters. One of the most prominent instantiations of the function is as follows. Given a query *Q *containing keywords *q*_1_,...,*q*_*n*_, the BM25 score of a document *D *is:

(1) Score(D,Q)=∑i=1nidf(q)i⋅tf(q)i⋅(k+11)tf(q)i+k⋅1(1−b+b⋅dlavdl)

where *tf*(*q*_*i*_) is *q*_*i*_'s term frequency in the document *D*, *dl *is the length of the document *D *in words, and *avdl *is the average document length in the text collection from which documents are drawn. *k*_1 _and *b *are free parameters, usually chosen as *k*_1 _= 2.0 and *b *= 0.75. *idf*(*q*_*i*_) is the *idf *weight of the query term *q*_*i*_. It is usually computed as: *idf*(*q*_*i*_) =log(N−n(q)i+0.5n(q)i+0.5) where *N *is the total number of documents in the collection, and *n*(*q*_*i*_) is the number of documents containing *q*_*i*_.

### Authority Flow Ranking in Web Search

IR techniques view each document as an independent entity to discover relevant pages and ignore the link structure of the document collection. Quality of results could be improved by also considering link structure to discover relevant pages [6]. We refer to these link-based approaches as *authority flow ranking *which gained popularity with PageRank [7], where a global score is assigned to each Web page. PageRank was the key novelty behind the first Google Web search engine. Intuitively, a page is important (high PageRank) if it is pointed via hyperlinks from other important pages. This recursive definition leads to an iterative formula, as shown in Equation 2 below. Let *G*(*V*, *E*) be a graph, with a set of nodes (pages) *V *= {*v*_*1*_, ..., *v*_*n*_} and a set of edges (hyperlinks) *E*. A surfer starts from a random node *v*_*i *_of *V*. At each step, the surfer follows a hyperlink with probability *d *or concludes that relevance is low and so randomly jumps to another node with probability 1 - *d*. The PageRank value of *v*_*i *_is the probability *r*(*v*_*i*_) that at a given time, the surfer is at *v*_*i*_. If there are *n *pages, and we denote by ***r ***the vector [*r*(*v*_*1*_), ..., *r*(*v*_*i*_), ..., *r*(*v*_*n*_)]^T ^then we have

(2) $$r=dAr+\frac{\text{(1 - d)}}{\text{|V|}}.e$$

where ***A ***is a *n *× *n *matrix with ***A***_ij _= 1OutDeg(v)j if there is an edge *v*_*j*_→*v*_*i *_in *E *and 0 otherwise, where *OutDeg*(*v*_*j*_) is the outgoing degree of node *v*_*j*_. Furthermore, *e *= [1, ..., 1]^T^. Notice that the vector *e *acts as a rank source, the set of nodes where the surfer jumps when concluding that relevance is low. In the original PageRank [7], all pages in the web graph act as rank sources. Note that PageRank measures the global importance of the pages independent of a keyword query. It has been found that 0.85 is a good value for *d *[7]. The CO ranking algorithm is an extension of PageRank to account for the various entity types and also create a query-specific and not global ranking of the entities. Note that an entity can be defined in many ways, e.g., a whole patient EHR can be viewed as a single entity, or individual components of the EHR like physicians, hospitalizations, medications, can be viewed as entities. The success of authority-flow algorithms depends on the choice of the entities' definition. In our experiments, we roughly create an entity for each tuple of the relational database, e.g., patient, hospitalization, and so on. In the case of XML, we could create an entity for each top-level XML element.

Haveliwala [8] proposes a topic-sensitive PageRank, where the topic-specific PageRanks for each page are pre-computed and the PageRank value of the most relevant topic is used for each query. Brinkmeier [9] models pagerank as a power series (in contrast to the usual Markov Chain modeling) and presents some results concerning the convergence of the standard iteration used for PageRank. Lin [10] discusses the application of PageRank like algorithms to related document networks comprised of automatically-generated content-similarity links (instead of manually-created hyperlinks) to prove the applicability of link analysis algorithms to different environments. Shepelyansky et. al [11] discusses an approximation of PageRank that is more efficient, by creating directed networks constructed by Ulam method with characteristics being rather similar to those of the World Wide Web. All of the above described works apply to the Web and do not address the unique characteristics of structured databases. In particular, the web graph has only one entity type (the web page) and one relationship type (the hyperlink between related web pages). In contrast, a structured database could have a variety of entity types and relationships [12] (e.g., in the EHR dataset of the cardiovascular division of Miami Children's Hospital there are relationships like patient-to-hospitalization, hospitalization-to-exam, and so on).

### Keyword Search in Databases and ObjectRank

While PageRank applies authority flow ranking on World Wide Web (WWW) which is largely unstructured, EHRs are typically semi-structured (XML format) or completely structured (relational format). A tiny fraction of the Web uses the semi-structured XML format (HTML tags are mostly for presentation purposes, and hence do not generally define semantic entities). Note that this work cannot be applied to unstructured EHRs, e.g., a collection of narrative medical reports, where no links exists between data entities. Health standards organizations like Health Level 7 have been designing XML-based formats to represent EHRs. Such XML formats have been adopted by pioneering health institutes like Regenstrief [13], and variations of these formats are expected to be adopted by many more institutes in the near future. XRANK [14] applies authority flow ranking over XML document collections. We use ObjectRank [12,15], which applies keyword query based authority flow ranking techniques on structured relational databases, as our experimental dataset is a relational anonymized EHR database. In contrast to PageRank, ObjectRank can find relevant pages that do not contain the keyword, but are directly linked from pages that do. Other works [16,17] perform non-authority-based keyword search on databases, where the focus is on finding connections between the query keywords and non on finding the most relevant entities.

We next give an overview of ObjectRank [12,15], which is the base of our CO system. In contrast to PageRank, instead of using the whole set of nodes *V *as rank sources (called *base set S *in ObjectRank), i.e., the set of nodes where the surfer jumps when concluding that relevance is low, ObjectRank uses an arbitrary subset *S *of nodes, thereby increasing the authority associated with the nodes of *S *and the ones most closely associated with them. The base set is set to contain the nodes that contain at least one of the query keywords. In particular, they define a *base vector ****s ***= [*s*_*0*_, ..., *s*_*i*_, ..., *s*_*n*_]^*T *^where *s*_*i *_is 1 if *v*_*i *_∈ *S *and 0 otherwise. The ObjectRank equation is then given as follows:

(3) $$r=dAr+\frac{\text{(1 - d)}}{\text{|S|}}\cdot s$$

Regardless of whether one uses Equation 2 or Equation 3, the PageRank algorithm solves this recursive equation using a simple iterative method, where the values of the (*k*+1)-th execution are calculated as follows:

(4) $$r^{(}{}^{\text{k}+\text{1})}=dAr^{k}+\frac{\text{(1 - d)}}{\text{|S|}}\cdot s$$

The algorithm terminates when ***r ***converges, which is guaranteed to happen under common conditions [18]. In particular, the authority flow graph needs to be irreducible (i.e., (*V*, *E*) be strongly connected) and aperiodic. The former is true due to the damping factor *d*, while the latter happens in practice. In addition to using a query-specific base set, the second innovation of ObjectRank is the weighing of the various associations types, e.g., patient to hospitalization vs. hospitalization to medication.

*ObjectRank Example: *Consider the damping factor *d *= 0.85 and query *Q *= ["*pericardial effusion*"], on the clinical data subset shown in Figure 1. Note that in ObjectRank, the *base set S *= {*v1*, *v4*, *v5*} has all objects that contain "*pericardial effusion*". In this example,

$$A=\left[\begin{array}{ccccccc} 0.0 & 0.0 & 0.0 & 0.0 & 0.0 & 0.0 & 0.0\\ 0.0 & 0.0 & 0.5 & 0.0 & 0.0 & 0.0 & 0.0\\ 0.0 & 0.0 & 0.0 & 0.0 & 0.0 & 0.0 & 0.0\\ 0.0 & 0.0 & 0.0 & 0.0 & 0.0 & 0.0 & 0.0\\ 0.0 & 0.0 & 0.0 & 0.0 & 0.0 & 0.0 & 0.0\\ 0.0 & 0.0 & 0.5 & 0.5 & 1.0 & 0.0 & 0.0\\ 1.0 & 0.0 & 0.0 & 0.5 & 0.0 & 1.0 & 0.0 \end{array}\right]\text{and }s={\left[\text{1},0,0,\text{1},\text{1},0,0\right]}^{T}\;\left|\text{S}\right|=\text{3}.$$

The computed ObjectRank scores vector *r *= [0.05,0.00,0.00,0.05,0.05,0.063,0.117]^T ^after one iteration. These scores indicate the importance and relevance of the entities in the clinical dataset in Figure 1 for query "pericardial effusion". The higher the score, the more relevant and important the entity is for the query. tf*idf scores rank entities by considering them individually, while ObjectRank scores enhance these rankings by also considering the relationships between them.

ObjectRank can be applied to clinical data provided the data is semi-structured (XML format) or structured (relational format) with some minor adaptations. The choice of entities is critical, given that it determines the authority flow and the ranking. Also, CO parameters must be calibrated to fit the application needs.

In our past work on authority flow we have proposed the ObjectRank ranking paradigm for bibliographic data [12,15]. In this paper we apply for the first time this paradigm on health data. For that, we created new ranking variants, as described below, to better suit this domain. Our recent book [1] on information discovery on EHRs briefly discusses the possibility of applying authority flow techniques on EHRs, but without specific algorithms or experiments.

### Data Modelling

**Figure 2:**
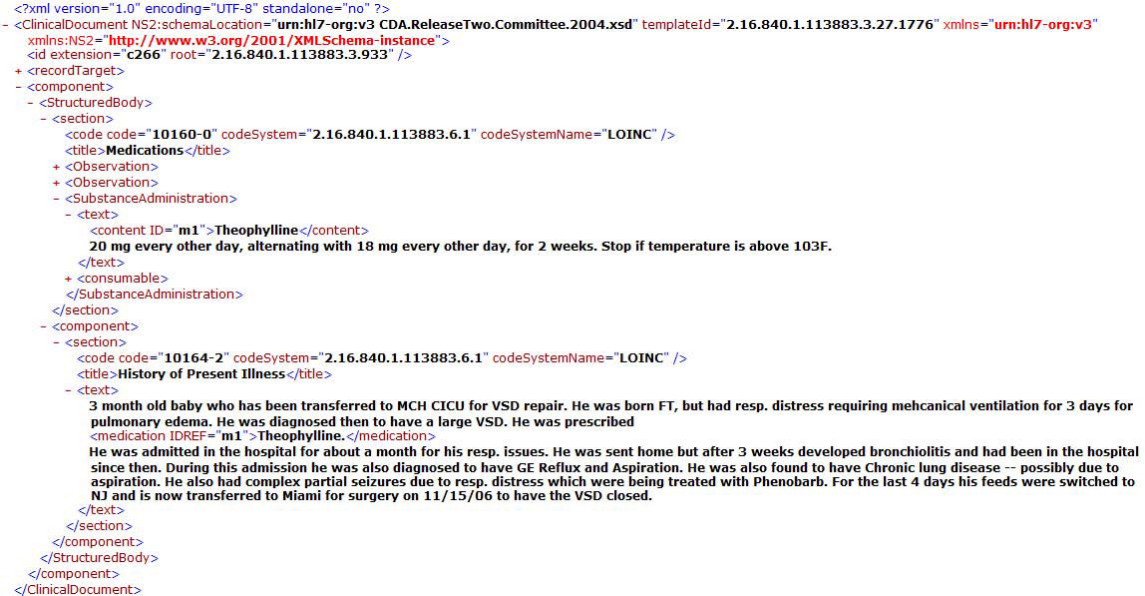
**An example HL7 CDA XML medical document**. An example HL7 CDA XML medical document that shows the use of ID/IDREF attributes in XML.

The fundamental difference between the two search methods studied here, BM25 and CO, stems from their different modelling of the collection of EHRs: in BM25, every document is modelled as a bag of keywords. CO models the corpus as a graph of interconnected entities. In particular, the dataset is viewed as a graph where each entity is modelled as a node and edges denote associations among various entities, such as a link from patient to hospitalization or from hospitalization to medication. This data model can abstract for both XML and relational data. Health standards organizations like Health Level 7 have been designing XML-based formats to represent EHRs. The Clinical Document Architecture [19] is such a format. An XML document can be represented as a hierarchical tree of nodes under a unique root element. In this model every XML element is represented as a node, and the parent-child relationships between elements are captured as edges called *containment *edges. The use of ID/IDREF attributes in XML [14] creates an additional edge--the *ID/IDREF *edge--between elements that are not directly connected by a parent-child relationship. This introduces cycles and hence transforms the tree into a graph. Figure 2 shows a medical record in HL7 CDA format. In this example, there is an element in the document with an ID-type attribute: <content ID="m1">Theophylline</content> and elsewhere, there is another element that refers to it: <medication IDREF="m1"> ...</medication>.

Likewise, relational data can also be modelled as a graph where relational entities are viewed as nodes, and primary-foreign key relationships (e.g., hospitalization to patient or medication to hospitalization) between the entities are represented as edges in the graph.

A *data graph D*(*V*, *E*) is a labelled directed graph where every node *v *has a label λ(*v*) and a set of keywords. For example, the node *v1 *of Figure 1 has label "*Events Plan*" and the set of keywords {''*residual*, ''*pericardial effusion''*, ''*2004-11-03*"}. Each node represents an *object *of the database and contains excerpts which are attribute values of the object in the source relational database. CO assumes that each node has a set of attribute name-value pairs. For example, the "*Hospitalization*" nodes of Figure 1 have *TimeStampCreated*, *history*, and *allergies *attributes. The keywords appearing in the attribute values comprise the set of keywords associated with the node. Each node *v *has a role λ(*v*). For instance, node *v3 *in Figure 1 has role "*Medication*". Each edge *e *from *u *to *v *is labelled with its role λ(*e*) and represents a relationship between *u *and *v*. For example, every "*medication*" to "*employee*" edge of Figure 1 has the label "*prescribed_by*". We manually pick edge role names. However, the names of the edge roles are not important in the algorithms execution. Figure 1 presents a subset of our clinical data graph *D*(*V,E*).

**Figure 3:**
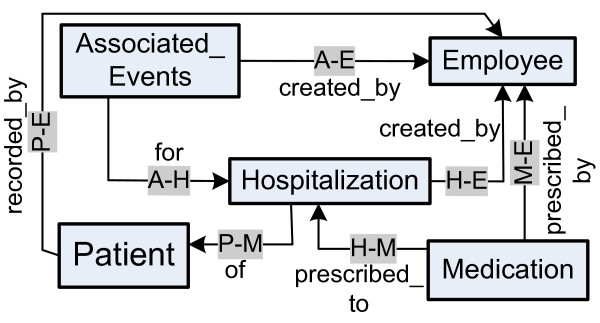
**Schema of the EHR dataset**. The schema graph of the EHR dataset is a directed graph that describes the structure of the EHR dataset in Figure 1.

The *schema graph G*(*V*_*G*_,*E*_*G*_) (Figure 3) is a directed graph that describes the structure of a data graph *D*(*V,E*). Every node has an associated label. Each edge is labelled with a role, which may be omitted. For instance, the *associated_events*-to-*employee *edge in Figure 3 has role "*created_by*".

### Clinical Data Mining and Medical Ontologies

Data mining differs from searching, since data mining techniques look for interesting patterns or trends in the data, whereas search algorithms look for relevant discrete pieces of data in a collection, given a query. Since data mining is not the focus of this work, we give some further pointers to readers who might be more interested in this area. Works on data mining in the clinical domain include [20-25]. Further, there has been recent work on the use of medical ontologies to improve the search quality, where the ontology is generally used to perform query expansion [26-28]. However, the use of ontologies is orthogonal to the use of authority flow, that is, authority flow can be used to rank the entities of the dataset and their association to the ontology or traditional IR can be used to rank the entities of the dataset and their association to the ontology.

### Clinical ObjectRank (CO) Variants

As mentioned before, in the original PageRank paper [18], the damping factor *d *was set to 0.85. The damping factor *d *determines the portion of ObjectRank that an object transfers to its neighbors as opposed to keeping itself. Changing the damping factor *d *offers another calibration opportunity. In particular, larger values of *d *favor nodes pointed by high-authority nodes through single-edge or multi-edge paths, while smaller values of *d *favor nodes containing the actual keywords--nodes in the base set--or their immediate neighbors. In other words, smaller *d *leads to more focused results. Before conducting a user survey, we tested several values of the damping factor in the range [1] and found that two values - 0.3 and 0.85 - gave reasonable results. Also, we wanted to test a smaller and a higher damping factor for reasons mentioned before. In particular, we considered the following variants of CO:

***CO085***: CO with *d *= 0.85. This is the default CO variant. Given that *d *is high, a result may not contain the query keywords, if many entities that contain the keywords link to the result.

***CO030***: CO with *d *= 0.30. As mentioned above, small *d *favours focused results that contain the actual query keywords.

***CO085BM25***: CO with *d *= 0.85 is combined with BM25. The top-*k *results were produced by multiplying CO scores with BM25 scores.

### Experimental Evaluation

The experimental dataset is a subset of the relational anonymized EHR database of the Cardiac Division of Miami Children's Hospital, which contains clinical entities like *hospitalization*, *patient*, *employee, medication*, *diagnosis, diagnostics, cardiac, events*, and *labs*. The dataset has been anonymized by replacing the names by sequential string ids (Patient1, Patient2, and so on), the dates by a relative count of seconds from the first timestamp, and also by manually removing all personal references in the text of the EHRs. To maximize performance in our testing environment, the survey uses only a subset of the EHR dataset with timestamps between June 2005 and December 2005. The dataset contains a mix of inpatient and outpatient events and is modeled as a clinical web with entities interconnected with one another based on their relationships. The hospitalization objects are inpatient events. A batch of SQL queries was executed on the source relational database to extract this experimental subset of the EHR dataset with timestamps between June 2005 and December 2005. The dataset contains a total of 15,092 clinical entities (nodes), of which 850 were hospitalization objects, and 31,492 were relationships (edges). Notice that the rest of the 14,242 entities are non-hospitalization ones comprising of patient, employee, medication, diagnosis, diagnostics, cardiac, events, and lab records.

### Pilot User Study

A pilot user study was conducted to compare the effectiveness, by evaluating the quality of results produced by two different techniques for keyword search in EHRs: traditional BM25 technique and CO -- a newly proposed authority flow ranking technique. In particular, to evaluate the quality of the results of each approach, we computed the mean sensitivity and specificity of the results produced by them. In our experiments, we use a relational anonymized EHR database provided by the Cardiac Division of Miami Children's Hospital. As we see in Figure 3, the schema links through primary-to-foreign keys entities like patients, hospitalizations of patients, employees, medications and associated events. The latter may be various clinical tests of patients or any other action.

During an initial version of the User Survey that we conducted, we found that the raters are mostly interested in Hospitalization entities as they provide very specific details of an inpatient event. The rest of the entity types (the non-hospitalization ones) - patient, employee, medication, diagnosis, diagnostics, cardiac, events, and labs, assist in the ranking of the hospitalization entities. Hence, we computed the score of each entity for each query and then filtered all non-hospitalization entities, since the purpose of our survey is to retrieve and rank only hospitalization objects. This makes the comparison between CO and BM25 fairer. Notice that the filtering step is very trivial as it simply involves removing non-hospitalization entities from the result list and is not part of the search method since it is just a post-processing step conducted after the results are ranked.

We performed a user study to compare the BM25 and CO methods for ranking the results of queries on a collection of EHRs. The users were asked to select the top 5 results that were both relevant and important for each query. These relevance/non-relevance ratings of users for the results of various ranking algorithms were used to compute specificity and sensitivity scores. Further, we propose a method for "*explaining*" the query results to the users, that is, speculate about why certain results were ranked higher than others.

**Figure 4:**
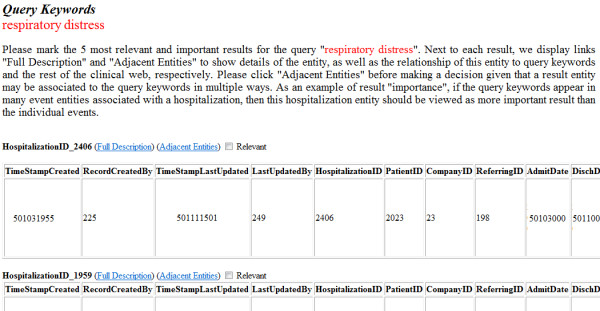
**Sample User Survey Page for query "*respiratory distress*"**. Figure 4 shows a sample user survey page for query "*respiratory distress*". It starts with a brief explanation about the survey and then query results are displayed. Each result displays a brief description of the clinical entity in tabular format along with links to the complete description and adjacent entities.

**Figure 5:**
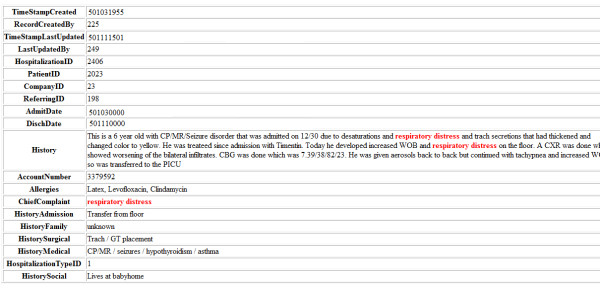
**Sample "Full Description" of HospitalizationID 2406 for query "*respiratory distress*"**. Figure 5 shows the Full Description of HospitalizationID_2406 for query "*respiratory distress*" in tabular format with each row displaying an attribute-value. Each occurrence of the query phrase is highlighted.

To gain preliminary insights about CO vs. BM25, we worked with a convenience sample of two primary-care physicians (MW and PB), who were blinded to the automated rankings. Note that the users have been trained before since we also conducted an initial version of the User Survey for the users to get used to the way the survey was built. In addition to this, we also allowed users to play with a demonstration website that permitted them the key in any set of keywords and see the full description of results along with the corresponding explaining sub graph of the result. For each keyword query, the top 5 results from each algorithm (BM25, CO085, CO030, and CO085BM25) were computed and merged to a single list of results for that query. The information about results and the corresponding algorithms was hidden from the users. Then, for each keyword query, the user was asked to select the top 5 results that were both relevant and important for each query. To help the users evaluate the results, next to each result we displayed the links "*Full Description*" and "*Adjacent Entities*", to show the details of the entity, as well as the relationship of this entity to query keywords and the rest of the clinical web, respectively. Figure 4 shows a sample user survey page for query "*respiratory distress*". Figure 5 shows a sample "*Full Description*" page of a hospitalization object for query "*respiratory distress*".

**Figure 6:**
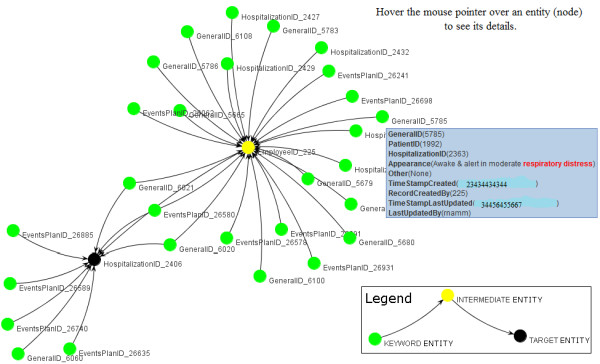
**Sample *explaining sub graph *of an ObjectRank2 result - HospitalizationID 2406 for query "*respiratory distress*"**. Figure 6 displays an explaining sub graph for HospitalizationID 2406 for query "*respiratory distress*". The figure gives a better picture of why this entity was ranked higher for query "*respiratory distress*" and its relationship with other entities that contain the query keywords. Detail descriptions of each entity are displayed as tool tip texts when the user points at them.

We briefly describe what happens when the user clicks the "*Adjacent Entities*" link, which "explains" the result. We believe that when complex ranking methods like BM25 and CO are employed, the users need information about why certain results are ranked high. Varadarajan *et al*. [29] described a novel technique to explain the results of authority-flow based techniques. For both CO and BM25 methods, we provide an "adjacent entities" link that pictorially explains each result. In the case of CO, an explaining graph is displayed using the principles described in [29]. For BM25, a similar explaining graph is displayed by including all entities that neighbor the target entity and also contain the query keywords. Figure 6 shows the explaining graphs, as shown in our user interface for CO. It might be easy to just view the Figure as a sub graph where the result entity is the central part surrounded by the neighbouring entities which can either contain the query keywords, thus making them relevant, or, could just act as intermediate entities helping connect the keyword entities with the result entity. For better clarity, we use colour coding to differentiate the entities, as can be seen in Figure 6.

A set of keyword queries was supplied by a group of physicians, as shown in Additional file 1: **Table s1**. We also show the containment statistics for these query terms.

### Evaluation Measures

To evaluate the results of each approach, we computed the sensitivity and specificity of each method, for each query. The physician users defined the truth or gold standard.

## Results

Additional file 1**: Table s1 **presents the sensitivity and specificity for each query and algorithm variant. All CO variants outperform BM25 in terms of sensitivity, while achieving similar specificity. Additional file 2: **Table s2 **presents the sensitivity and specificity of individual raters for each query and algorithm variant. The different raters seem to agree on the relevance/usefulness of CO085BM25 and CO030 results as the amount of disagreement was trivial (the 2 raters had almost same sensitivity/specificity values for these 2 variants) but largely disagreed on the usefulness of CO085 and BM25 results (the 2 raters had significant differences in their sensitivity/specificity values for these 2 variants). A reason for the variance among users is that users can choose to see or not see the explaining graph for each result, which may bias their relevance judgment. Despite the differences in ratings, this pilot study shows that ObjectRank results are more valued (i.e., selected) by the raters than BM25 results.

## Discussion

For many types of automated searching of EHRs, maintaining a high sensitivity is important. For example, identifying patients who may meet criteria for a certain treatment or preventive measure first requires identifying all potentially eligible patients and only then can lead to applying exclusion criteria. In this study, in a limited sample of test cases, CO provided, on average, a 71% improvement over BM25. This difference was observed without a compromise in specificity. The CO030 variant performed best, in terms of both sensitivity and specificity. Thus, authority-flow techniques have potential to improve information discovery in structured EHRs. Note that CO030 has damping factor *d *= 0.3, which is different from the standard *d *= 0.85 used in Web search. The reason may be that the purpose of PageRank on the Web is to create a global ranking, whereas in CO we create a query-specific ranking, and hence the authority should not flow far from the entities that contain the actual query terms. Recall that smaller *d *leads to shorter flow of authority in the data graph.

We considered possible explanations as to why CO's sensitivity varied among the queries. In simplest terms, the sequence of keywords that define a query might be contained in an result record, neighboring records, or both result and neighboring records. Users are more likely to give a higher rank to results containing the query phrase compared to results where only neighbouring linked records contain the query phrase. For example, this appeared to be true for query #3 (data not shown). In contrast, the presence of the query phrase in multiple neighboring records could lead to a higher CO ranking than a query phrase found only in a result record. This appeared true in query #11 (data not shown), for which CO had high sensitivity. This difference between human and CO approaches likely led to the variable sensitivity. We would infer in many such cases that the human gold standard may have actually been faulty in failing to detect certain relevant cases. Indeed, our own reviews and discussions about the findings suggested this.

In testing and discussions, we found that some queries may be so vague as to generate unhelpful results. For example, a query of "Tetralogy of Fallot" is medically specific, whereas a query of "edema" can assume so many meanings--such as peripheral edema, pulmonary edema, and cerebral edema--that identifying all records containing the word "edema" may be, in retrospect, unwanted. Thus, when it comes to crafting a useful product for IR, even a sensitive one can unlikely compensate for a poorly formulated query.

We also analyzed how much agreement occurred between raters and what might account for differences between raters. Overall, the cases of differences are ones where the query phrase is often absent from the core description but may be represented in various combinations of nodes. Many of the differences in ratings seem to reflect somewhat subtle variations in how the raters subjectively assess the importance of the nodes. An opportunity for the future might then be "tuning" or appending to the algorithm, to reflect individual users' weights or priorities in how the ranking is done (e.g., relative importance of query phrase vs. other terms, core description vs. nodes). Despite differences in ratings, many of the additional results found with ObjectRank but not BM25 are valued (i.e., selected) by the raters.

### Limitations

This study has limitations. The performance was evaluated with only two end users. This was done by design so that we could gain preliminary insights into performance and discuss possible explanations about differences between the methods. A larger sample would clearly be needed to make more definitive conclusions. The dataset reflects pediatric cases, so we cannot be certain that the results would be the same in other datasets, populations, or institutions. Nevertheless, we did not identify any particular qualities in the dataset that would suggest such a strong limitation.

Applying the CO ranking paradigm to other domains or datasets has several challenges. First, the appropriate entities must be identified. For instance, should a patient be in the same entity as her hospitalization? The choice of entities is critical, given that it determines the authority flow graph and the ranking. Second, the CO parameters must be calibrated. In particular, the damping factor *d *and the authority transfer rates must be calibrated. Our recent work [29] proposes a technique for the system to learn the authority transfer by exploiting the user relevance feedback, that is, the user's "click-through" pathway. Via customization, various CO profiles can be created to suit the diverse information needs of various kinds of users, such as researchers, pharmacists, physicians, and nurses. For example, for a pharmacist, the medications are typically most important; for a researcher, the patient and employee information may not be as relevant as other information. We can achieve customization by weighing relationships differently for various profiles.

## Conclusions

In comparison of two fundamentally different techniques for keyword search in an EHR system--the traditional Information Retrieval BM25 technique and an authority flow ranking technique, CO--all CO variants outperform BM25 in terms of sensitivity, while achieving similar specificity. Future investigation should use larger sample sizes to confirm CO's advantages.

## Competing interests

The authors declare that they have no competing interests.

## Authors' contributions

VH conceived the whole study, participated in its design and coordination and helped to draft the manuscript. RRV carried out the implementation of Clinical ObjectRank (CO), BM25 systems and User Survey and drafted the manuscript. PB and MW participated in the user survey, analyzed results, contributed to conclusions and discussions and helped to draft and revise the manuscript. All authors read and approved the final manuscript.

## Pre-publication history

The pre-publication history for this paper can be accessed here:

http://www.biomedcentral.com/1472-6947/10/64/prepub

## Supplementary Material

Additional file 1 **Table s1 - Average Sensitivity and Specificity of Algorithm Variants**. Table 1 displays the sensitivity and specificity of each survey query for different algorithm variants along with some statistics about the queries. The average sensitivity and selectivity values of each algorithm variant are presented at the end. Click here for file

Additional file 2 **Table s2 - Sensitivity and Specificity of algorithms with respect to individual raters**. Table 1 displays the individual sensitivity and specificity values of the two raters for all survey queries for different algorithm variants. Click here for file
